# The relationship between teachers’ zest for work and teaching motivation: the mediating role of achievement goals

**DOI:** 10.3389/fpsyg.2024.1362920

**Published:** 2024-06-27

**Authors:** Alper Aytaç, Çiğdem Şahin, Deniz Görgülü, Yaşar Dilber, Ayhan Direk

**Affiliations:** ^1^Ministry of National Education, Bursa, Türkiye; ^2^Ministry of National Education, Adana, Türkiye; ^3^Ministry of National Education, Konya, Türkiye; ^4^Ministry of National Education, Düzce, Türkiye

**Keywords:** zest for work, achievement goals, teaching motivation, teachers, education sociology, Türkiye

## Abstract

**Objective:**

This study aims to investigate the mediating role of achievement goals in the relationship between teachers’ zest for work and teaching motivation.

**Method:**

The research was designed using the relational survey model. The research sample consisted of 518 teachers working in various cities in Turkey in 2023–2024 academic year fall semester. A convenience sampling method was used in sampling. Three Likert-type scales were used as data collection tools. In the data analysis, firstly, the data suitability to normal distributions was examined. As a result of the analysis, kurtosis and skewness values were examined and it was assumed that the data were normally distributed. SPSS Process extension was used to analyze the data.

**Results and discussion:**

According to the research results, teachers’ zest for work positively and significantly predicted strong and positive achievement goals. In addition, teachers’ achievement goals significantly and positively predicted their teaching motivation. Additionally, teachers’ zest for work positively and significantly predicted their teaching motivation. Moreover, it can be inferred that achievement goals for students have a mediating role in the relationship between teachers’ zest for work and teaching motivation. In this context, it is suggested that policies that increase teachers’ teaching motivation should be prioritized.

## 1 Introduction

For Concerns about the quality of countries’ public education systems are drawing attention to key elements of teacher effectiveness (Darling-Hammond, 2000). When the elements presented to determine teacher effectiveness are examined, the level of knowledge and skills that the teacher brings to the classroom and the demonstrated achievement in classroom practices are shown as important variables (Lewis et al., 1999). However, teachers’ attitudes and behaviors in the classroom are also among the most critical factors in creating permanent behavioral changes in students – which is a main goal of education (Goe, 2002; İhtiyaroğlu, 2018). In this context, it can be stated that teachers’ attitudes and behaviors in the teaching process are of great importance. Pleasure, which is defined as approaching life with hope, excitement and energy and is also a strong positive feature of teaching (Peterson and Seligman, 2004), is considered an important trait that has an impact on teachers’ behaviors (Hoy et al., 2006).

Teachers’ zest for work is considered an effective element in increasing students’ motivation (Kunter et al., 2011). Teachers’ zest for work is also effective in increasing the quality of instruction and education (Weström et al., 2018). In addition, teachers’ zest for work can also be used as a criterion in evaluating the education process (Peterson and Park, 2006). In this respect, it can be argued that teachers’ zest for work is related to their achievement goals for students. A high level of focus on the work is deemed necessary for the flow (Sahranç, 2021), which is among the concepts related to zest for work and described as the state of being motivated for a long time (Yaşin, 2016). In this context, it can be deduced that a teacher’s zest for work and teaching motivation are highly related. In addition, according to the ERG (Existence, Relatedness, Growth) Theory, which is a motivational theory from Clayton Alderfer who simplified Maslow’s hierarchy of needs. One of the most basic needs is for individuals to be successful (Alderfer, 1969). For this reason, it is possible to motivate employees with achievement (Özkalp and Kırel, 2011). When it is considered from the teaching perspective, motivation is as important as skills and abilities and is related to openness to professional development (Butler, 2007; Watt and Richardson, 2007).

One of the primary tasks of educational institutions is to achieve predetermined institutional goals. Achieving these goals depends on many variables. Teachers are one of the most influential in achieving the goals set in the education and training process. Fulfilling professional roles, achieving strategic goals and providing quality education are closely related to teachers’ zest for work and teaching motivation. In addition to motivation, it has been determined that teachers’ high levels of zest for work can have positive effects on both their own lives and their school lives (Erdoğan Ş., 2013). Also, it is seen that zest for work and motivation are in a close relationship with increased and improved students’ achievement. In this context, the research aimed to reveal the mediating role of achievement goals in the relationship between teachers’ zest for their work and students’ motivation to learn. Before this review, these concepts were briefly discussed.

## 2 Literature review

### 2.1 Zest for work

Zest for work is defined as the satisfaction an individual feels from his or her job. An individual with a high zest for work is often expected to have positive attitudes and behaviors regarding his/her profession (Yazıcı et al., 2013). Zest for work is a concept that includes enthusiasm and stronger emotions in addition to a feeling of job satisfaction. However, it contains more vitality and enjoyment of life than job satisfaction (Tüfekçi, 2015). Teachers’ zest for life is viewed as their emotional reactions and subjective attitudes towards their job or teaching role. This refers to the functional relationship between one’s instructional expectations and what is actually delivered (Skaalvik and Skaalvik, 2011).

Toropova et al. (2021) state that happier teachers tend to have happier students, and more satisfied teachers provide higher quality instruction to their students. Evidence has shown that zest for work is a psychological state as well as an emotional experience that people have. It can affect the job itself, salary, promotion, ability to handle job pressures, and interpersonal relationships with leaders and colleagues in the work environment (Dou et al., 2017; Wang et al., 2021). Higher levels of zest for work among teachers are noted to promote effective teaching and learning (Muguongo et al., 2015). While zest for work at the individual level affects teachers’ job enthusiasm and mental health, from the school management perspective, teachers’ zest for work is seen as a factor affecting teaching, school quality, and the efficiency of school leadership (Hongying, 2007).

In educational environments, teachers’ professional satisfaction is of vital importance. Teachers’ zest for work is strongly related to work engagement, a measure of intrinsic motivation (Klassen et al., 2012). Teachers who are intrinsically motivated and enjoy their work typically report having more job resources, which increases their overall well-being and sense of satisfaction in their work (Bakker and Bal, 2010). Additionally, an effective working environment, personal development, teachers’ enthusiasm for their work, and psychological well-being are also linked to job engagement (Lucas-Mangas et al., 2022). In addition, teachers’ zest for work increases students’ willingness to work, revealing the possible effects of teachers’ participation in their work on student achievement (Bradbury and Wilson, 2020).

It has been found that teachers’ zest for work can provide numerous educational benefits for both schools and students. It can create a positive atmosphere of trust, improve school performance, increase collegiality, raise teaching motivation and improve overall achievement (Toropova et al., 2021). Both the self-development opportunities which each job provides, and job satisfaction efforts for an individual may differ. Examining teachers’ zest for work among professions should have a special importance because teachers’ educating and raising future generations is a difficult process that often requires superior effort (Koruklu et al., 2013).

### 2.2 Teaching motivation

Motivation is a critical issue for schools as it affects many different aspects of the educational environment. In educational environments, motivation is a subject that attracts more and more attention as shown by a significant increase in the number of related studies published in recent years. It is a focus of attention in both schools and academic environments (Ryan and Deci, 2016; Patall, 2021).

Motivation is explained as a person’s taking action with his/her own efforts and will in order to achieve personally and also uphold and improve the ideals of the school (Ugar, 2019). From an organizational perspective, motivation is a process that encourages individuals to take action to achieve organizational goals (Oco, 2022). Motivation can also lead to actions taken that motivate a person towards a desired goal. Motivation is a powerful concept that affects an individual’s needs, goals, desires, level of satisfaction, level of participation and selection and engagement in interests (Başaran and Güçlü, 2018).

An individual who is intrinsically motivated wants to make the work interesting and enjoyable (Aslan and Doğan, 2020) does not expect rewards (grades, money, etc.) and his/her behavior stems mainly from personal drive and inspiration (Topçuoğlu-Ünal and Bursalı, 2013). This also applies to teachers.

Teachers’ teaching motivation, which is considered as important as their teaching abilities (Güzel-Candan and Evin-Gencel, 2015), is one of the most important factors that affect teaching-learning processes positively or negatively (De Jesus and Lens, 2005). Individuals with an extrinsic motivation towards teaching seek financial gain, etc. Teachers with intrinsic teaching motivation carry out their activities without expecting any reward, are often more patient, and continue their work with a personal satisfaction-oriented approach (Kauffman et al., 2011). Motivated teachers tend to support their students’ motivation in their quest for meeting basic psychological needs such as autonomy, competence and belonging (Abós et al., 2018).

Because of its complex effects on a range of teaching and learning activities, teacher motivation is a crucial component in educational environments. First of all, teachers’ motivation has a great impact on their professional commitment, their well-being, and happiness (Mahler et al., 2018). Teachers who have professional commitment are more likely to be motivated, which creates a healthy work environment and ultimately impacts student outcomes. Additionally, according to Haakma et al. (2017), there is a strong correlation between teachers’ motivation and the learning environments they create. Motivated educators are more likely to use successful teaching techniques and create a warm, stimulating learning environment in the classroom. All this has a positive impact on students’ motivation and academic success. In addition, teachers’ motivation is also of great importance in retaining excellent educators within the institution.

### 2.3 Achievement goals

Achievement goals are defined as “the goal or cognitive-dynamic focus of competency-related activities.” Goal orientation is thought to cause the individual to develop an understanding of how to describe, interpret, and respond to the achievement environment (e.g., work, sports, and school) (Pintrich, 2000). Achievement goals are closely related to how individuals define and interpret the concept of success in educational environments. Teachers work to attain their own achievement goals and outcomes in educational environments (Butler, 2007). Achievement goals also enable teachers to promote and develop learning outcomes in students. Teachers who focus on strong and useful achievement goals encourage student development and mastery of skills and abilities (Turner, 2014). Papaioannou and Christodoulidis (2007) examined teachers’ achievement goals through a three-factor structure, which they defined as learning goals (acquiring and developing professional competence), performance approach goals (demonstrating a superior teaching competence than others), and performance avoidance purposes (avoiding demonstrating a lower teaching competence than others).

Mascret et al. (2015) explains teachers’ achievement goals as follows:

- The aim of approaching the task is to ensure and support the students’ success,
- The aim of task avoidance is to prevent students from failing and experiencing failure,
- The aim of getting closer to the essence is to provide a better and more effective education,
- The aim of self-avoidance is to avoid worse and less effective teaching,
- the aim of getting closer to others is to provide better teaching than others,
- The aim of avoiding others is to avoid worse teaching than others.

Achievement goals are important for educators because they provide a framework for understanding teacher motivation and how it affects instructional strategies. In this context, achievement goal theory can help understand the relationship between the concepts in question. According to achievement theory, people’s motivations and achievement-related actions can be understood by considering their motivation or goal to accomplish a task (Zusho and Clayton, 2011). According to Butler (2012), achievement goal theory has been proposed as a potentially useful paradigm for understanding motivation for both teaching and learning. The theory emphasizes the importance of setting a variety of achievement goals and how these goals influence instructional strategies. In this context, according to Butler (2007), achievement goal theory can be used to understand teaching motivation as well as learning. This means that understanding instructors’ motivation and classroom behavior requires awareness of their achievement goals. In addition, Butler and Shibaz (2008) stated that achievement goal theory offers a potential framework for understanding teacher motivation and emphasized that although teachers have common desires to be successful in their careers, they have different teaching achievement goals. This highlights how important it is to recognize the diversity of achievement goals that teachers have and how these goals may influence their teaching strategies.

### 2.4 Relationships between zest for work, teaching motivation, and achievement goals

In the literature, teachers’ zest for work and achievement goals for students are considered as interrelated concepts. It is seen that zest for work has an impact on the psychological well-being of teachers (Büyükgöze, 2023), which in turn has a decisive effect on teachers’ achievement goals for students (Sahito and Vaisanen, 2020). It is noteworthy that this relationship also has some effects on student success (Hughes et al., 2008; Marsh and O’Mara, 2008; Freiberger et al., 2012; Han et al., 2019). It is thought that student success may have a significant and positive effect on the teacher’s teaching motivation. Research shows that teachers with high zest for work are more successful in their schools (Hayati and Caniago, 2012; Dreer, 2021; Admiraal et al., 2023; Meredith et al., 2023).

High teaching motivation, which is related to achievement goals for students, should be one of the most important objectives for schools. A teacher’s low teaching motivation will negatively affect the quality and quantity of his/her work and will also make it difficult for the school to achieve its educational goals (Padalia and Nurochmah, 2022). If teachers have a higher teaching motivation to realize achievement goals, they will often strive to do better. The teacher will often carry out the education and instruction process more seriously and with fewer difficulties. They will achieve higher success as a result. Teachers with high achievement goals often motivate students to more easily overcome the problems they encounter in the learning process and therefore better fulfill their duties as educators (Rival and Mulyadi, 2012). In this context, it can be inferred that there is a positive relationship between teachers’ achievement goals and teaching motivation.

The achievement goal approach has been used to explain the relationship between teachers’ teaching motivation and achievement goals in the educational environment (Daumiller et al., 2021). Butler and Shibaz (2008) revealed the relationships between teachers’ achievement goals and their attitudes. This review provides support in establishing a new understanding in terms of teaching motivation. However, in the research conducted by Butler (2007), examining the structure of teachers’ achievement goals is considered an important step in evaluating the understanding of teacher motivation. In general, zest and motivation are often considered words with very close ties in their meanings. But according to Hersey et al. (2001), these concepts are quite different from each other. While motivation expresses how willing individuals are to work to achieve the organizational goals (Li et al., 2014), zest for work often relates to the feelings that people have after receiving their material and/or moral rewards. In this respect, zest for work can be evaluated as a result of the immediate events experienced or engaged in by the individual in the organization. In motivation, there is an expectation for the future (Beekhan, 2012). On the other hand, research shows that there is a positive relationship between employees’ zest for work and motivation (Kreitner and Kinicki, 2001; Sirota et al., 2006; Thomason, 2006; Zhao and Jeon, 2023).

The efficient functioning of the education system is realized by the implementation of the curriculum in accordance with the original mandates (Hollins, 1996; Barnett, 2000). It is important that teachers, who are responsible for the implementation of curriculum in the learning-teaching process, are committed to the curriculum (Kimpston, 1985). One of the factors affecting teachers’ commitment to curriculum is their teaching motivation (Aytaç, 2021). In this regard, revealing which factors affect teachers’ teaching motivation is important for the efficiency of the education system. From a theoretical perspective, it is possible to say that individuals who are happy to fulfill their profession will be more motivated towards improving their profession.

Understanding the relationships between teachers’ teaching motivations, zest for work, and achievement goals is crucial to grasping the dynamics of the learning environment. Research has shown a strong relationship between teachers’ motivation and achievement goals and their levels of zest for work, sometimes known as “work enthusiasm.” For example, Toropova et al. (2021) emphasized the importance of teacher qualifications and school working conditions in determining zest for work, which is closely linked to teachers’ motivation and enjoyment of their work. Additionally, Salanova et al. (2006) examined the interaction between individual characteristics and work engagement in secondary school teachers by examining the relationship between personal and organizational resources and work-related flow. Peterson et al. (2009) also suggested that connections can be described as somewhat stimulating for work.

In this context, in this research, it is thought that teachers’ zest for work may have an impact on their teaching motivation. In addition, another concept related to individuals’ motivational processes is achievement goal orientation (Toros and ve Koroç, 2005). Achievement goals relate to competence and participation in achievement behavior (Mascret et al., 2015). Teachers’ achievement goals relate to efficiently achieving the goals delineated in the curriculum of the learning-teaching process. In this context, this research was built on the basic question ‘Do achievement goals have a mediating role in the relationship between teachers’ zest for work and teaching motivation?’ and four hypotheses were created in this direction.

When the related literature is examined, studies focusing on the relationships between these variables are quite limited, and no study examining these three variables together has been found. In this respect, this study has an originality that can contribute to the relevant literature. It is thought that examining the mediating role of achievement goals in the relationship between teachers’ zest for work and teaching motivation is important in terms of directly increasing the success of teachers and students, and indirectly helping schools achieve goals and increase effectiveness.

This research will contribute to understanding whether or not achievement goals for students play a mediating role in the relationship between teachers’ teaching motivation and zest for work. In addition, it is anticipated that the research results will provide a strong logical framework that can be added to the relevant literature and provide guidance for similar research. In practice, this research will contribute to issues such as (a) improving teacher effectiveness and development in teacher education programs in terms of motivation, zest for work, and designing achievement goals for students, and (b) encouraging teachers to determine strong and meaningful learning goals that will support their professional development.

## 3 Research method

### 3.1 Model

In this research, a relational survey method was used. According to Karasar (2006), relational survey models are used to reveal possible relationships between variables. In this study, the relational survey model was adopted because the mediating role of achievement goals in the relationship between teachers’ zest for work and teaching motivation was examined.

### 3.2 Population and sample

The population of this research consists of teachers working in various cities in Turkiye in the fall semester of the 2023–2024 academic year. The research sample consists of 518 teachers selected by a convenience sampling method. Convenience sampling method is an advantageous method in terms of speeding up the data collection process of the research. By continuing to work with a sample group that is easily accessible, the researcher saves time and has the opportunity to expand the sample as much as possible (Balcı, 2001; Özen and Gül, 2007). In this study, teachers who were easily accessible to the researchers and who worked in various cities constituted the sample. According to official statistics, there are over one million teachers in Türkiye (Ministry of National Education, 2023). According to the sample table stated by Cohen et al. (2000), it can be said that the number of samples in this research is sufficient within the 95% confidence interval. Information about the research sample is presented in Table 1.

**Table 1:** Data regarding the research sample.

Variable		Frequency (f)	Percentage (%)
Gender	Female	307	59.3
	Male	211	40.7
Professional seniority (year)	0–5	66	12.7
	6–10	71	13.7
	11–15	117	22.6
	16–20	103	19.9
	21 and above	161	31.1

In Table 1, the research sample was demonstrated. According to the Table 1, 307 of the participants are female (59.3%) and 211 (40.7%) are male. 66 (12.7%) of the participants have professional seniority between 0 and 5 years, 71 (13.7%) have between 6 and 10, 117 (22.6%) have between 11 and 15, 103 (19.9%) have between 16 and 20, and 161 (31.1%) have 21 years or more of professional seniority.

### 3.3 Data collection tools

Three scales were used as data collection tools in the research: Zest for Work Scale, Achievement Goals Scale, and Teaching Motivation Scale.

#### 3.3.1 Zest for work scale

The scale was developed by Erdoğan O. (2013). The sample of the scale development study consisted of 600 teachers. The theoretical basis of the scale was found by Peterson and Seligman (2004) and created a scale that measures the character traits of individuals. As a result of the Exploratory Factor Analysis (EFA), a single-dimensional structure consisting of 7 items was revealed. As a result of the Confirmatory Factor Analysis (CFA) performed, it was determined that the fit index were within acceptable ranges. Additionally, CFA was performed with this research data. Modifications were made to the analyses. According to the results, it is possible to say that the fit indexes are within acceptable ranges (CMIN/DF = 3.556, RMSEA = 0.026, GFI = 0.952, CFI = 0.985, NFI = 0.979, RMSEA = 0.070) (Sümer, 2000). The standardized diagram is presented in Appendix 2. The scale is a 5 point Likert type. As a result of the reliability analysis, the Cronbach alpha value was calculated as 0.890. As a result of the reliability analysis conducted within the scope of this research, the Cronbach alpha value was calculated as 0.885.

#### 3.3.2 Achievement goals scale

The scale was developed by Papaioannou and Christodoulidis (2007) and adapted to Turkish by Demirtaş and Arslan (2018). Two hundred and thirty-five teachers participated in the scale adaptation study. According to the results of the Confirmatory Factor Analysis (CFA), it was determined that the fit indexes of the scale were within acceptable ranges. The scale consists of three subdimensions and 12 items. Additionally, CFA was performed with this research data. Modifications were made to the analyses. According to the results, it is possible to say that the fit indexes are within acceptable ranges (CMIN/DF = 3.111, RMSEA = 0.064, GFI = 0.979, CFI = 0.969, NFI = 0.979, RMSEA = 0.064) (Sümer, 2000). The diagram is presented in Appendix 4. The scale is a 5-point Likert type. As a result of the reliability analysis, the Cronbach alpha value was calculated as 0.730. As a result of the reliability analysis conducted within the scope of this research, the Cronbach alpha value was calculated as 0.811.

#### 3.3.3 Teaching motivation scale

The scale was adapted by Kauffman et al. (2011) and adapted into Turkish by Güzel-Candan and Evin-Gencel (2015). The first stage of the scale adaptation process was translation the scale into Turkish and language control. At this stage, support was received from academics who are experts in the related field. After language control, the scale form was conducted to 342 teacher candidates. As a result of the Confirmatory Factor Analysis (CFA), it was determined that the fit indices of the scale were within acceptable ranges. The scale consists of two sub-dimensions and 12 items. Additionally, CFA was performed with this research data. Modifications were made to the analyses. According to the results, it is possible to say that the fit indexes are within acceptable ranges (CFI = 0.901, IFI = 0.902, GFI = 0.902) (Sümer, 2000). The diagram is presented in Appendix 6. The scale is 5 point Likert type. As a result of the reliability analysis, the Cronbach alpha values of the sub-dimensions, and the scale in total were calculated as 0.90, 0.79, 0.92. As a result of the reliability analysis conducted within the scope of this research, Cronbach alpha values were calculated as 0.79, 0.74, 0.88.

### 3.4 Data collection and analysis

Necessary permissions were obtained for the use of the scales before data collection. The research data was prepared using the Google forms database and then shared with the participants. Participation was based on volunteering. Later, the data were transferred to an SPSS Program. At this stage, first the data compliance with normality assumptions was examined. Descriptive statistics and normality values of the data are stated in Table 2.

**Table 2:** Descriptive statistics and coefficients of skewness-kurtosis.

Variables	*N*	Minimum	Maximum	$\bar{X}$	SD	Kurtosis	Skewness
ZW	518	1.43	5.00	4.08	0.74	0.576	−0.920
AG	518	1.75	5.00	3.59	0.64	−0.309	0.178
TM	518	1.00	6.00	3.52	1.02	−0.046	−0.182

According to the results in Table 2, it was determined that the kurtosis and skewness values of the data were within acceptable ranges (George and Mallery, 2010). Additionally, outlier analysis was performed. The data was examined for multicollinearity. If there is a high relationship between variables, it is possible to think about multicollinearity problem (Çokluk et al., 2014). In this regard, the results of Pearson correlation analysis among the variables were taken into consideration. The highest value obtained as a result of the correlation analysis between the variables was found to be 0.533. According to Büyüköztürk (2007), this value is at a medium level. As a result, it is possible to say that there is no multicollinearity problem between the variables.

**Figure 1:**
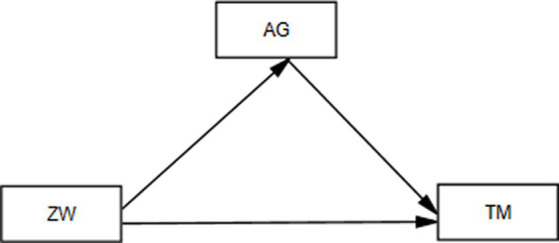
Path diagram. ZW, Zest for work; AG, achievement goals; TM, teaching motivation.

The research measurement model was analyzed through the SPSS Process extension. The measurement model of the research is presented in Figure 1. SPSS V3.5 version was used to calculate the mediating effect.

*H*₁ = Teachers’ zest for work positively and significantly predicts their achievement goals.

*H*₂ = Teachers’ professional achievement goals significantly predict their teaching motivation in a positive way.

*H*₃ = Teachers’ zest for work positively and significantly predicts their teaching motivation.

*H*₄ = Achievement goals have a mediating role in the relationship between teachers’ zest for work and teaching motivation.

## 4 Findings

This section provides findings for testing the model established to reveal the mediating role of achievement goals in the relationship between teachers’ zest for work and teaching motivation. First of all, simple linear regression analyzes were applied between the variables. The results of the regression analysis performed in this context are presented in Table 3.

**Table 3:** Regression analysis results between the variables.

Dependent variable: achievement goals (AG)						
Independent variable	*F*	*p*-value	*B*	S.E.	*t*	*p*-value
ZW	63.305	0.000	0.284	0.036	7.956	0.000
*R* = 0.331	*R*^2^ = 0.109					

Dependent variable: teaching motivation (TM)						
Independent variable	*F*	*p*-value	*B*	S.E.	*t*	*p*-value
AG	102.310	0.000	0.646	0.064	10.115	0.000
*R* = 0.407	*R*^2^ = 165					
ZW	205.144	0.000	0.729	0.051	14.323	0.000
*R* = 0.533	*R*^2^ = 284					

According to the results of the simple linear regression analysis in Table 3, teachers’ zest for work positively and significantly predicted their achievement goals (*B* = 0.284, *p* < 0.01). Teachers’ achievement goals positively and significantly predicted their teaching motivation (*B* = 0.646, *p* < 0.01). Teachers’ zest for work positively and significantly predicted their teaching motivation (*B* = 0.729, *p* < 0.01). As a result, the first three hypotheses of this research were accepted. Based on these results, the mediating effect discussed in the fourth hypothesis of the research can be examined in line with Baron and Kenny's (1986) theory.

In the second stage of the research, mediator effect analysis was carried out. For this purpose, first of all, the predictive effect of teachers’ zest for work and achievement goals on their teaching motivation was examined. This information is presented in Table 4.

**Table 4:** The predictive effect of zest for work and achievement goals on teaching motivations.

Independent variable	*F*	*p*-value	*B*	S.E.	*t*	*p*-value	LLCI	ULCI
ZW	135.090	0.000	0.612	0.052	11.843	0.000	0.511	0.714
AG			0.411	0.060	6.843	0.000	0.293	0.529
*R* = 0.587	*R*^2^ = 0.344							

According to the Table 4, teachers’ zest for work and achievement goals together significantly predict their teaching motivation (*F* = 135.090, *p* < 0.01). LLCI and ULCI values are between 0 and 1. According to Preacher and Hayes (2008), in order to reveal meaningful results, LLCI and ULCI value ranges must be less than or greater than zero. In this respect, it is possible to say that these results are meaningful. It is noteworthy that after the achievement goals were included in the regression model, the non-standard effect value of teachers’ zest for work on their teaching motivation decreased from 0.729 to 0.612. This may indicate that achievement goals may have a mediating role in the predictive effect of teachers’ zest for work on their teaching motivation. According to Holmbeck (1997), if the direct meaningful relationship between the independent variable and the dependent variable loses its significance after the mediator variable was engaged, a full mediating effect is possible. However, if the direct significant relationship between the independent variable and the dependent variable decreases and maintains its significance after the mediator variable engages, then a partial mediating effect can be mentioned.

In this study, the predictive effect of teachers’ zest for work on their teaching motivation remains significant and decreases slightly after the achievement goals variable is introduced. In this context, it is possible to conclude that achievement goals have a partial mediating effect on the predictive effect of teachers’ zest for work on their teaching motivation. As a result, the fourth hypothesis of the research was accepted.

## 5 Discussion

In this study, the mediating role of achievement goals in the relationship between teachers’ job zest for work and teaching motivation was examined. In this context, the research was designed on four hypotheses. These hypotheses are discussed, respectively.

Within the scope of the first hypothesis of the research, the predictive effect of teachers’ zest for work on their achievement goals was examined. As a result of the analysis, it was determined that teachers’ zest for work had a significant predictive effect on their achievement goals. In other words, the teachers’ zest for work is an important variable in explaining their achievement goals. Based on this result, it is possible to say that teachers who are happy to practice their profession and have a high level of zest for work can positively focus on increasing the success of the learning-teaching process. According to Büyükgöze and Özdemir (2017), there is a positive significant relationship between teachers’ zest for work and professional performance. In other words, the result of this research reveals that teachers who are happy to practice their profession have a high level of performance in the learning-teaching process. In Karaçam (2018) research, it was found that teachers’ zest for work affected their perception of achievement. Additionally, according to Toropova et al. (2021), it is emphasized that teachers’ zest for work supports the learning-teaching atmosphere to increase students’ success.

Within the scope of the second hypothesis of the research, the predictive effect of teachers’ achievement goals on their teaching motivation was examined. As a result of the analysis, it was determined that teachers’ achievement goals had a positive and significant predictive effect on their teaching motivation. In other words, the achievement goals are important variables in explaining teachers’ teaching motivations. Butler (2012) research also reveals the relationship between teachers’ achievement goals and their teaching motivation and approaches. The research finds out that teachers’ achievement goals are important for an effective teaching environment. According to Rival and Mulyadi (2012), teachers who are success-oriented in the learning-teaching process have high motivation. As a result, the basis of the achievement goal approach is the relationship between teachers’ achievement goals and teaching motivation in the educational environment (Daumiller et al., 2019). In a longitudinal study conducted with faculty members, Daumiller et al. (2023) found out that achievement goals are effective on faculty members’ burnout levels and performance. Fokkens-Bruinsma et al. (2018) revealed that there is a significant relationship between teachers’ motivation for their work and their students’ achievement goals.

Within the scope of the third hypothesis of the research, the predictive effect of teachers’ zest for work on their teaching motivation was examined. As a result of the analysis, it was determined that teachers’ motivation had a positive and significant predictive effect on their zest for work. In other words, the teachers’ zest for work is an important variable in explaining their teaching motivation. The research results conducted in the relevant literature also point to the existence of a relationship between the zest for work and motivation of working individuals (Kreitner and Kinicki, 2001; Sirota et al., 2006; Thomason, 2006; Zhao and Jeon, 2023). According to Skaalvik and Skaalvik (2011), teachers’ zest for work affects their teaching motivation. Niu et al. (2023) state that teachers’ professional pleasure and job satisfaction positively affect their motivation for teaching. Amin (2015) revealed in the research that there is a positive relationship between teachers ‘job satisfaction and teachers’ teaching motivation.

Within the scope of the fourth hypothesis of the research, the mediating role of achievement goals in the predictive relationship between teachers’ zest for work and teaching motivation was examined. An analysis determined that achievement goals had a partial mediator role in the predictive relationship between teachers’ zest for work and their teaching motivation. In other words, achievement goals also have an impact on the predictive effect of teachers’ zest for work on their teaching motivation. The results of this research show that zest for work has both a direct and indirect effect on teaching motivation. As teachers’ achievement goals increase, the effect of their zest for work on their teaching motivation increases. According to Schunk et al. (2014), individuals who are goal-oriented towards achievement are more willing to improve both themselves, others, and their environment. The individuals in this situation also increase their motivation during the actions they take (Lemyre et al., 2002; Wolters, 2004). This supports he results that showed that achievement goal orientations are an important factor in explaining the motivational processes of teachers with high levels of zest for work. Teaching motivation is mainly explained by three factors: personal efficacy, interest in the task, and effort spent on teaching responsibilities (Almeida et al., 2021). Binda and Koloi-Keaikitse (2020) emphasized that teachers’ zest for work and teaching experience are effective on students’ academic success, and that students’ academic performance is an important predictor of teachers’ zest for work and teacher’s teaching experience.

## 6 Conclusion and suggestions

In this study, the mediating role of achievement goals in the relationship between teachers’ zest for work and teaching motivation was examined and four hypotheses were determined in this context. The analyses showed all four hypotheses were accepted. Positive and significant predictive relationships were determined between teachers’ zest for work, achievement goals and teaching motivation. The relational screening model showed achievement goals have a mediating role but only partial. Other variables may also have a mediating effect on the relationship between teachers’ zest for work and teaching motivation. This presents an important challenge and research topic for researchers working in the relevant field. Theoretical readings can be made regarding which variables can be included in this model as mediator variables.

The research results provide important implications for those who manage education policies. It is important for the learning-teaching process that teachers have a high level of teaching motivation. The necessary experiences must be supported so that teachers’ zest for work in designing achievement goals for students are at a high level. In this context, school administrations can also be involved in the process to ensure that teachers’ working environments have a positive atmosphere.

## Data availability statement

The raw data supporting the conclusions of this article will be made available by the authors, without undue reservation.

## Ethics statement

The studies were conducted in accordance with the local legislation and institutional requirements. Ethical review and approval was not required for the study on human participants in accordance with the local legislation and institutional requirements. The participants provided their written informed consent to participate in this study. The authors declare that this study is an original study; that we have acted in accordance with the principles and rules of scientific ethics at all stages of the study, including preparation, data collection, analysis and presentation of information; that we have cited sources for all data and information not obtained within the scope of this study and included these sources in the bibliography; that we have not made any changes in the data used; and that we comply with ethical duties and responsibilities by accepting all the terms and conditions of the Committee on Publication Ethics (COPE).

## Author contributions

AA: Formal analysis, Methodology, Software, Writing – original draft, Writing – review & editing. CS: Conceptualization, Supervision, Writing – original draft, Writing – review & editing. DG: Supervision, Writing – original draft, Resources. YD: Supervision, Writing – review & editing. AD: Writing – review & editing.

## References

[ref1] Abós Á. Haerens L. Sevil J. Aelterman N. García-González L. (2018). Teachers’ motivation in relation to their psychological functioning and interpersonal style: a variable-and person-centered approach. Teach. Teach. Educ. 74, 21–34. doi: 10.1016/j.tate.2018.04.010

[ref2] Admiraal W. Kittelsen Røberg K. I. (2023). Teachers’ job demands, resources and their job satisfaction: satisfaction with school, career choice and teaching profession of teachers in different career stages. Teach. Teach. Educ. 125, 104063–104010. doi: 10.1016/j.tate.2023.104063

[ref3] Alderfer C. P. (1969). An empirical test of a new theory of human needs. Organ. Beha. Hum. Perform. 4, 142–175. doi: 10.1016/0030-5073(69)90004-X

[ref4] Almeida L. S. Moreira M. A. Caldeira S. N. Soares S. M. Hattum-Janssen N. V. Visser-Wijnveen G. J. (2021). Teachers’ motivation for teaching in higher education: Portuguese validation of a questionnaire. Paidéia 31, 1–11. doi: 10.1590/1982-4327e3104

[ref5] Amin M. (2015). Relationship between job satisfaction, working conditions, motivation of teachers to teach and job performance of teachers in MTs, Serang, Banten. J. Manag. Sustain. 5, 141–154. doi: 10.5539/jms.v5n3p141

[ref6] Aslan M. Doğan S. (2020). A theoretical view of extrinsic motivation, intrinsic motivation and performance interaction. Süleyman Demirel Univ. Vision. J. 11, 291–301. doi: 10.21076/vizyoner.638479

[ref7] Aytaç A. (2021). A study of teachers’ self-efficacy beliefs, motivation to teach, and curriculum fidelity: a path analysis model. Int. J. Contemp. Educ. Res. 8, 130–143. doi: 10.33200/ijcer.898186

[ref8] Bakker A. B. Bal P. M. (2010). Weekly work engagement and performance: a study among starting teachers. J. Occup. Organ. Psychol. 83, 189–206. doi: 10.1348/096317909x402596

[ref9] Balcı A. (2001). Sosyal bilimlerde araştırma: Yöntem, teknik ve ilkeler. Ankara: PegemA Yayıncılık.

[ref10] Barnett R. (2000). Supercomplexity and the curriculum. Stud. High. Educ. 25, 255–265. doi: 10.1080/713696156

[ref11] Baron M. Kenny D. A. (1986). The moderator-mediator variable distinction insocial psychological research: conceptual, strategic and statistical considerations. J. Pers. Soc. Psychol. 51, 1173–1182. doi: 10.1037/0022-3514.51.6.1173 3806354

[ref12] Başaran M. Güçlü N. (2018). Analyzing the relationship between school principles’ management styles and teachers’ job satisfaction. Gazi Univ. J. Gazi Educ. Facult. 38, 949–963. doi: 10.17152/gefad.402996

[ref13] Beekhan A. (2012). The relationship between achievement motivation and job satisfaction. (Master of social science). South Africa: School of Psychology University of Kwazulu-Natal.

[ref14] Binda P. C. D. Koloi-Keaikitse S. (2020). Teacher job satisfaction and teacher experience as predictors of students’ mathematics performance. Mosenodi J. 23, 24–35.

[ref15] Bradbury L. Wilson R. (2020). Questioning the prevailing narrative about elementary science teachers: an analysis of the experiences of science teacher enthusiasts. Sci. Educ. 104, 421–445. doi: 10.1002/sce.21574

[ref16] Butler R. (2007). Teachers’ achievement goal orientations and associations with teachers’ help seeking: examination of a novel approach to teacher motivation. J. Educ. Psychol. 99, 241–252. doi: 10.1037/0022-0663.99.2.241

[ref17] Butler R. (2012). Striving to connect: extending an achievement goal approach to teacher motivation to include relational goals for teaching. J. Educ. Psychol. 104, 726–742. doi: 10.1037/a0028613

[ref18] Butler R. Shibaz L. (2008). Achievement goals for teaching as predictors of students’ perceptions of instructional practices and students’ help seeking and cheating. Learn. Instr. 18, 453–467. doi: 10.1016/j.learninstruc.2008.06.004

[ref19] Büyükgöze H. (2023). Job satisfaction with profession among teachers in Türkiye: perceptions of social utility and educational policy ınfluence. Participat. Educ. Res. 10, 200–213. doi: 10.17275/per.23.82.10.5

[ref20] Büyükgöze H. Özdemir M. (2017). Examining job satisfaction and teacher performance within affective events theory. İnönü Univ. J. Facult. Educ. 18, 311–325. doi: 10.17679/inuefd.307041

[ref21] Büyüköztürk Ş. (2007). Sosyal bilimler için veri analizi el kitabı. Ankara: PegemA Yayıncılık.

[ref22] Cohen L. Manion L. Morrison K. (2000). Reserch methods in education. London: Routledge/Falmer.

[ref23] Çokluk Ö. Şekercioğlu G. Büyüköztürk Ş. (2014). Sosyal bilimler için çok değişkenli istatistik SPSS ve LISREL uygulamaları. Ankara: Pegem Akademi.

[ref24] Darling-Hammond L. (2000). Teacher quality and student achievement: a review of state policy evidence. Educ. Policy Analysis Archives 8:1. doi: 10.14507/epaa.v8n1.2000

[ref9001] Daumiller M. Dickhäuser O. Dresel M. (2019). University instructors’ achievement goals for teaching. J. Educ. Psychol. 111:131.

[ref25] Daumiller M. Fasching M. Dickhäuser O. Dresel M. (2023). Teachers’ achievement goals and teaching practices: a standardized lesson diary approach. Teach. Teach. Educ. 127:104079. doi: 10.1016/j.tate.2023.104079

[ref26] Daumiller M. Grassinger R. Engelschalk T. Dresel M. (2021). SEEQ-DE: construction and validation of a German adaption of the instrument "student evaluation of educational quality" (Marsh) [SEEQ-DE: Konstruktion und Überprüfung einer deutschsprachigen adaption des instruments “student evaluation of educational quality” (Marsh)]. Diagnostica 67, 176–188. doi: 10.1026/0012-1924/a000274

[ref27] De Jesus S. N. Lens W. (2005). An integrated model for the study of teacher motivation. Appl. Psychol. Int. Rev. 54, 119–134. doi: 10.1111/j.1464-0597.2005.00199.x

[ref28] Demirtaş Z. Arslan N. (2018). Teachers' achievement goals: a mixed method. Univ. J. Educ. Res. 6, 710–720. doi: 10.13189/ujer.2018.060414

[ref29] Dou D. Devos G. Valcke M. (2017). The relationships between school autonomy gap, principal leadership, teachers’ job satisfaction, and organizational commitment. Educ. Manag. Admin. Leadersh. 45, 959–977. doi: 10.1177/1741143216653975

[ref30] Dreer B. (2021). Teachers’ well-being and job satisfaction: the important role of positive emotions in the workplace. Educ. Stud. 50, 61–77. doi: 10.1080/03055698.2021.1940872

[ref31] Erdoğan O. (2013). İlköğretim öğretmenlerinin öz yeterlik ve başarı algılarında yordayıcı olarak akademik iyimserlik, umut ve mesleki haz. (Yüksek lisans tezi). Ankara: Gazi Üniversitesi.

[ref32] Erdoğan Ş. (2013). Örgüt iklimi ile çalışanların motivasyonu ve iş tatmini arasındaki ilişkiler: Özel bir hastanede uygulama. (Yüksek lisans tezi). İzmir: Gediz Üniversitesi Sosyal Bilimler Enstitüsü.

[ref33] Fokkens-Bruinsma M. Canrinus E. T. ten Hove M. Rietveld L. (2018). The relationship between teachers' work motivation and classroom goal orientation. Pedagogische Studiën 95, 86–100.

[ref34] Freiberger V. Steinmayr R. Spinath B. (2012). Competence beliefs and perceived ability evaluations: how do they contribute to intrinsic motivation and achievement? Learn. Individ. Differ. 22, 518–522. doi: 10.1016/j.lindif.2012.02.004

[ref35] George D. Mallery P. (2010). SPSS for windows step by step: A simple guide and reference 17.0 update. Boston: Pearson.

[ref36] Goe L. (2002). The distribution of emergency permit teachers in California. Educ. Policy Analysis Archives 10, 1–36. doi: 10.14507/epaa.v10n42.2002

[ref37] Güzel-Candan D. Evin-Gencel İ. (2015). Adaptation of the motivation to teach scale into Turkısh. Mehmet Akif Ersoy Univ. J. Facult. Educ. 36, 72–89.

[ref38] Haakma I. Janssen M. Minnaert A. (2017). The influence of need-supportive teacher behavior on the motivation of students with congenital deafblindness. J. Visual Impair. Blind. 111, 247–260. doi: 10.1177/0145482x1711100305

[ref39] Han H. Bong M. Kim S.-i. Kwon S. K. (2019). Utility value and emotional support of teachers as predictors of student utility value and achievement. Educ. Psychol. 42, 421–438. doi: 10.1080/01443410.2019.1693509

[ref40] Hayati K. Caniago I. (2012). Islamic work ethic: the role of intrinsic motivation, job satisfaction, organizational commitment and job performance. Procedia Soc. Behav. Sci. 65, 272–277. doi: 10.1016/j.sbspro.2012.11.122

[ref41] Hersey P. Blanchard K. H. Johnson D. E. (2001). Management of organizational behaviour: Leading human resources. 8th Edn. New Jersey: Prentice-Hall.

[ref42] Hollins H. R. (1996). Culture in school learning: revealing the deep meaning. Mahwah: L. Erlbaum.

[ref43] Holmbeck G. N. (1997). Toward terminological, conceptual, and statistical clarity in the study of mediators and moderators: examples from the child-clinical and pediatric psychology literatures. J. Count. Clin. Psychol. 65, 599–610. doi: 10.1037//0022-006x.65.4.599, PMID: 9256561

[ref44] Hongying S. (2007). Literature review of teacher job satisfaction. Chin. Educ. Soc. 40, 11–16. doi: 10.2753/CED1061-1932400502

[ref45] Hoy W. K. Tarter C. J. Hoy A. W. (2006). Academic optimism of schools: a force for student achievement. Am. Educ. Res. J. 43, 425–446. doi: 10.3102/00028312043003425

[ref46] Hughes J. N. Luo W. Kwok O. Loyd K. L. (2008). Teacher-student support, effortful engagement, and achievement: a 3-year longitudinal study. J. Educ. Psychol. 100, 1–14. doi: 10.1037/0022-0663.100.1.1, PMID: 19578558 PMC2705122

[ref47] İhtiyaroğlu N. (2018). Analyzing the relationship between happiness, teachers' level of satisfaction with life and classroom management profiles. Univ. J. Educ. Res. 6, 2227–2237. doi: 10.13189/ujer.2018.061021

[ref48] Karaçam A. (2018). Zest for work in physical education and sport teachers' perceptions of success. Asian J. Educ. Train. 4, 391–395. doi: 10.20448/journal.522.2018.44.391.395

[ref49] Karasar N. (2006). Bilimsel araştırma yöntemi: Kavramlar, ilkeler teknikler. Ankara: Nobel Yayınevi.

[ref50] Kauffman D. F. Yılmaz Soylu M. Duke B. (2011). Validatıon of the motivation to teach scale. H. U. J. Educ. 40, 279–290.

[ref51] Kimpston R. D. (1985). Curriculum fidelity and the implementation tasks employed by teachers: a research study. J. Curric. Stud. 17, 185–195. doi: 10.1080/0022027850170207

[ref52] Klassen R. M. Perry N. E. Frenzel A. C. (2012). Teachers' relatedness with students: an underemphasized component of teachers' basic psychological needs. J. Educ. Psychol. 104, 150–165. doi: 10.1037/a0026253

[ref53] Koruklu N. Feyzioğlu B. Özenoğlu-Kiremit H. Kaldırım E. (2013). Examining teachers job satisfaction level according to some variables. Mehmet Akif Ersoy Univ. J. Facult. Educ. 1, 119–137.

[ref54] Kreitner B. Kinicki A. (2001). Organizational behaviour. USA: Richard D. Irwin Inc.

[ref55] Kunter M. Frenzel A. Nagy G. Baumert J. Pekrun R. (2011). Teacher enthusiasm: dimensionality and context specificity. Contemp. Educ. Psychol. 36, 289–301. doi: 10.1016/j.cedpsych.2011.07.001

[ref56] Lemyre P. Roberts G. C. Ommundsen Y. (2002). Achievement goal orientations, perceived ability and sports personship in youth soccer. J. Appl. Sport Psychol. 14, 120–136. doi: 10.1080/10413200252907789

[ref57] Lewis L. Parsad B. Carey N. Bartfai N. Farris E. Smerdon B. (1999). Teacher quality: a report on the preparation and qualifications of public school teachers. Washington: Statistical Analysis Report.

[ref58] Li L. Hu H. Zhou H. He C. Fan L. Liu X.. (2014). Work stress, work motivation and their effects on job satisfaction in community health workers: a cross-sectional survey in China. BMJ Open 4:e004897. doi: 10.1136/bmjopen-2014-004897 PMC4054641 24902730

[ref9002] Lucas-Mangas S. Valdivieso-León L. Espinoza-Díaz I. M. Tous-Pallarés J. (2022). Emotional intelligence, psychological well-being and burnout of active and in-training teachers. Int. J. Environ. Res. Public Health 2022, 19:3514. doi: 10.3390/ijerph19063514 35329207 PMC8951300

[ref59] Mahler D. Großschedl J. Harms U. (2018). Does motivation matter? – the relationship between teachers’ self-efficacy and enthusiasm and students’ performance. PLoS One 13:e0207252. doi: 10.1371/journal.pone.0207252, PMID: 30462713 PMC6248951

[ref60] Marsh H. W. O’Mara A. (2008). Reciprocal effects between academic self-concept, self-esteem, achievement, and attainment over seven adolescent years: unidimensional and multidimensional perspectives of self-concept. Personal. Soc. Psychol. Bull. 34, 542–552. doi: 10.1177/0146167207312313, PMID: 18340036

[ref61] Mascret N. Elliot A. J. Cury F. (2015). Extending the 3 × 2 achievement goal model to the sport domain: the 3 × 2 achievement goal questionnaire for sport. Psychol. Sport Exerc. 17, 7–14. doi: 10.1016/j.psychsport.2014.11.001

[ref63] Meredith C. Moolenaar N. Struyve C. Vandecandelaere M. Gielen S. Kyndt E. (2023). The importance of a collaborative culture for teachers’ job satisfaction and affective commitment. Eur. J. Psychol. Educ. 38, 43–62. doi: 10.1007/s10212-022-00598-w

[ref64] Ministry of National Education. (2023). Milli eğitim istatistikler: Örgün eğitim. Available at: https://sgb.meb.gov.tr/www/icerik_goruntule.php?KNO=508

[ref65] Muguongo M. M. Muguna A. Muriithi D. K. (2015). Effects of compensation on job satisfaction among secondary school teachers in Maara Sub-County of Tharaka Nithi County, Kenya. J. Hum. Resour. Manag. 3:47. doi: 10.11648/j.jhrm.20150306.11

[ref66] Niu J. Fan C. Wang Z. Chen Y. (2023). Multi-level analysis of factors on teacher job satisfaction across Japan and South Korea: evidence from TALIS 2018. SAGE Open 13, 215824402311785–215824402311714. doi: 10.1177/21582440231178533

[ref67] Oco R. (2022). Level of job satisfaction of public high school teachers: a survey. Int. J. Res. Publ. 95, 114–133. doi: 10.47119/IJRP100951220222888

[ref68] Özen Y. Gül A. (2007). Sosyal ve eğitim bilimleri araştırmalarında evren-örneklem sorunu. Kazım Karabekir Eğitim Fakültesi Dergisi 15, 394–422.

[ref69] Özkalp E. Kırel Ç. (2011). Örgütsel davranış. Bursa: Ekin Yayınevi.

[ref70] Padalia A. Nurochmah A. (2022). The relationship of achievement motivation with teacher performance in the implementation. Teach. Learn. Process Senior High Schools 654, 1–5. doi: 10.2991/assehr.k.220402.001

[ref71] Papaioannou A. Christodoulidis T. (2007). A measure of teachers’ achievement goals. Educ. Psychol. 27, 349–361. doi: 10.1080/01443410601104148

[ref72] Patall E. A. (2021). Self-determination theory: eminent legacy with boundless possibilities for advancement. Motiv. Sci. 7, 117–118. doi: 10.1037/mot0000223

[ref73] Peterson C. Park N. (2006). Character strengths in organizations. J. Organ. Behav. 27, 1149–1154. doi: 10.1002/job.398

[ref74] Peterson C. Park N. Hall N. R. Seligman M. E. P. (2009). Zest and work. J. Organ. Behav. 30, 161–172. doi: 10.1002/job.584

[ref75] Peterson C. Seligman M. E. P. (2004). Character strengths and virtues: A handbook and classification. New York: Oxford University Press.

[ref76] Pintrich P. R. (2000). “The role of goal orientation in self-regulated learning” in Handbook of self-regulation. eds. Boekaerts M. Pintrich P. R. Zeidner M. (San Diego, CA: Academic Press), 451–502. Available at: https://psycnet.apa.org/doi/10.1016/B978-012109890-2/50043-3

[ref77] Preacher K. J. Hayes A. F. (2008). Asymptotic and resampling strategies for sssessing and comparing indirect effects in multiple mediator models. Behav. Res. Methods 40, 879–891. doi: 10.3758/BRM.40.3.879, PMID: 18697684

[ref78] Rival V. Mulyadi D. (2012). Leadership and organizational behavior. Jakarta: Eagle Press.

[ref79] Ryan R. M. Deci E. L. (2016). “Facilitating and hindering motivation, learning, and well-being in schools: research and observations from self-determination theory” in Handbook of motivation at school. eds. Wentzel K. R. Miele D. B. (New York, NY: Routledge), 96–119.

[ref80] Sahito Z. Vaisanen P. (2020). A literature review on teachers’ job satisfaction in developing countries: recommendations and solutions for the enhancement of the job. Rev. Educ. 8, 3–34. doi: 10.1002/rev3.3159

[ref81] Sahranç Ü. (2021). “Akış kuramı” in Eğitimde pozitif psikoloji uygulamalar. eds. Tekinalp B. E. Işık Ş. (Ankara: PegemA), 111–139.

[ref82] Salanova M. Bakker A. B. Llorens S. (2006). Flow at work: evidence for an upward spiral of personal and organizational resources. J. Happiness Stud. 7, 1–22. doi: 10.1007/s10902-005-8854-8

[ref83] Schunk D. Meece J. R. Pintrich P. (2014). Motivation in education: Theory, research, and applications. Boston: Pearson.

[ref84] Sirota D. Mischkind L. A. Meltzer M. I. (2006). Stop demotivating your employees. Harvard Management Update, 11:1.

[ref85] Skaalvik E. Skaalvik S. (2011). Teacher job satisfaction and motivation to leave the teaching profession: relations with school context, feeling of belonging, and emotional exhaustion. Teach. Teach. Educ. 27, 1029–1038. doi: 10.1016/j.tate.2011.04.001

[ref86] Sümer N. (2000). Yapısal eşitlik modelleri. Türk Psikoloji Yazıları 3, 49–74.

[ref87] Thomason T. R. (2006). ICU nursing orientation and post orientation practices: a national survey. Crit. Care Nurs. Q. 29, 237–245. doi: 10.1097/00002727-200607000-00008, PMID: 16862025

[ref88] Topçuoğlu-Ünal F. Bursalı H. (2013). Turkish teachers’ views about motivation factors Middle Eastern & African. J. Educ. Res. 10, 76–96. doi: 10.19128/turje.832203

[ref89] Toropova A. Myrberg E. Johansson S. (2021). Teacher job satisfaction: the importance of school working conditions and teacher characteristics. Educ. Rev. 73, 71–97. doi: 10.1080/00131911.2019.1705247

[ref9003] Toros T. ve Koroç Z. (2005). Hedef Yönelimleri ve Algılanan Motivasyonel İklim Arasındaki İlişki. Spor Bilimleri Dergisi, 16, 135–145.

[ref90] Tüfekçi B. (2015). Öğretmenlerin finansal iyi hal düzeylerinin mesleki haz düzeylerine etkisi (Kartal ilçesi örneği). (Yayımlanmamış yüksek lisans tezi) İstanbul: Okan Üniversitesi Sosyal Bilimler Enstitüsü Eğitim Bilimleri Anabilim Dalı Eğitim Yönetimi ve Denetimi Programı.

[ref91] Turner A. (2014). Elementary teachers' achievement goal orientations in a high-stakes accountability context: A validation study, (a dissertation submitted in partial fulfillment of the requirements for the doctor of philosophy in education). Virginia: Educational Psychology at Virginia Commonwealth University.

[ref92] Ugar Y. (2019). Okul müdürlerinin liderlik uygulamaları ile öğretmenlerin motivasyonu arasındaki ilişki. (Yayımlanmamış yüksek lisans tezi). İstanbul: İstanbul Sabahattin Zaim Üniversitesi Sosyal Bilimler Enstitüsü.

[ref93] Wang Y. Stein D. Shen S. (2021). Students’ and teachers’ perceived teaching presence in online courses. Distance Educ. 42, 373–390. doi: 10.1080/01587919.2021.1956304

[ref94] Watt H. M. G. Richardson P. W. (2007). Motivational factors influencing teaching as a career choice: development and validation of the FIT-choice scale. J. Exp. Educ. 75, 167–202. doi: 10.3200/JEXE.75.3.167-202

[ref95] Weström S. Uusiautti S. Maatta K. (2018). The force that keeps you going: enthusiasm in vocational education and training (VET) teachers work. Int. J. Res. Vocat. Educ. Train. 5, 244–263. doi: 10.13152/IJRVET.5.4.1

[ref96] Wolters C. A. (2004). Advancing achievement goal theory: using goal structures and goal orientations to predict students' motivation, cognition, and achievement. J. Educ. Psychol. 96, 236–250. doi: 10.1037/0022-0663.96.2.236

[ref97] Yaşin T. (2016). Kişilik özellikleri ve psikolojik sermayenin psikolojik iyi oluş, akış deneyimi, iş tatmini ve çalışan performansına etkileri (Yayımlanmamış Doktora Tezi). Sosyal Bilimler Enstitüsü, Ankara: Başkent Üniversitesi.

[ref98] Yazıcı H. Gamsız Ş. Altun F. (2013). Type a personality, stress resources, self-efficacy and job satisfaction among teachers. J. Turkish Stud. 8, 1475–1488. doi: 10.7827/TurkishStudies.5328

[ref99] Zhao X. Jeon L. (2023). Examining the associations between teacher job satisfaction, workplace climate, and well-being resources within head start programs. Early Educ. Develop. 1–17. doi: 10.1080/10409289.2023.2221765

[ref100] Zusho A. Clayton K. E. (2011). Culturalizing achievement goal theory and research. Educ. Psychol. 46, 239–260. doi: 10.1080/00461520.2011.614526

