# Supplementary material for: The relationship between teachers’ zest for work and teaching motivation: the mediating role of achievement goals
Source: Front Psychol. 2024 Jun 27;15:1362920. doi: 10.3389/fpsyg.2024.1362920 (PMC11236758; doi:10.3389/fpsyg.2024.1362920)

Appendix

Appendix 1. Zest for Work Scale Sample Items

- I would like to be a part of working life instead of watching it from the sidelines.
- I look forward to each new working day.
- When I start a project, I start without wasting time.
- I start the day feeling excited about the opportunities my job will bring.
- I love my job.
- I have a pleasant time at my workplace.

Appendix 2. Zest for Work Scale Confirmatory Factor Analysis Diagram

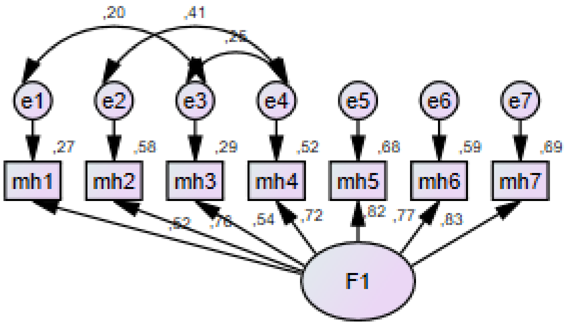

Appendix 3. Achievement Goals Scale Sample Items

- I would definitely be pleased to see that I am a better teacher than others.
- I avoid being ridiculed by methods beyond my ability.
- My goal is to constantly improve my skills as a teacher.
- I intend to work even harder to learn new things about what I teach
- I will continue to avoid exercises where I may appear inadequate
- It is important for me to always learn new things about the subject I teach

Appendix 4. Achievement Goals Scale Confirmatory Factor Analysis Diagram

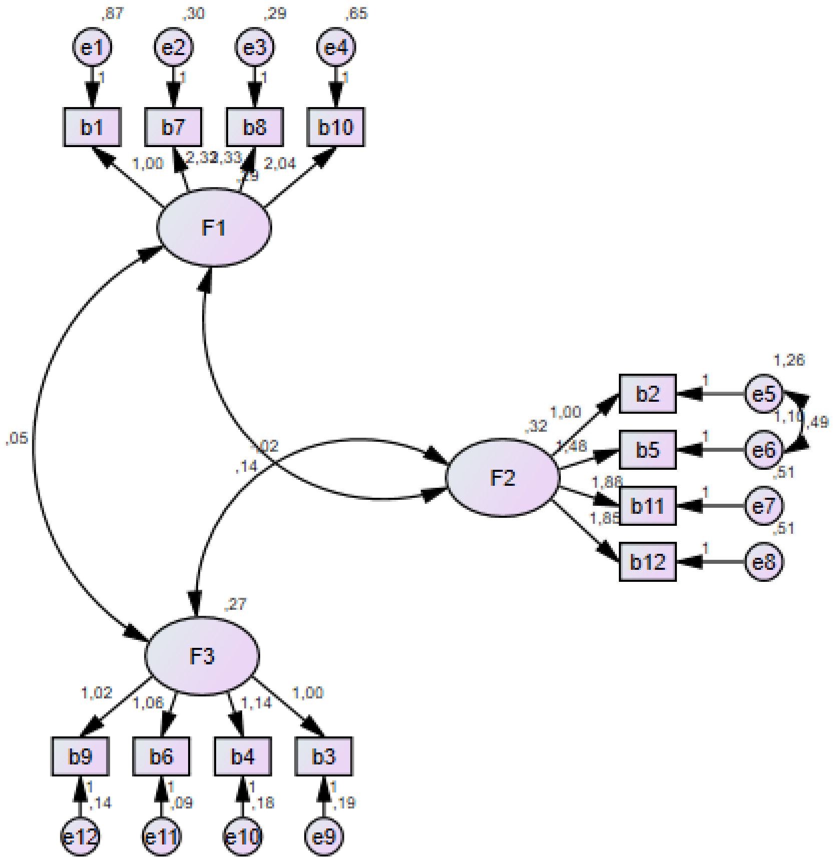

Appendix 5. Teaching motivation Scale Sample Items

- I chose teaching because it would help me get a better position in the future.
- I can't think of a more enjoyable career than teaching.
- I chose teaching because of the freedom it gave me.
- I chose teaching because a teaching degree would allow me to find a job almost anywhere.
- I get excited when I share my decision to become a teacher with others.
- I chose teaching because I would be respected in society as a teacher.

Appendix 6. Teaching Motivation Scale Confirmatory Factor Analysis Diagram

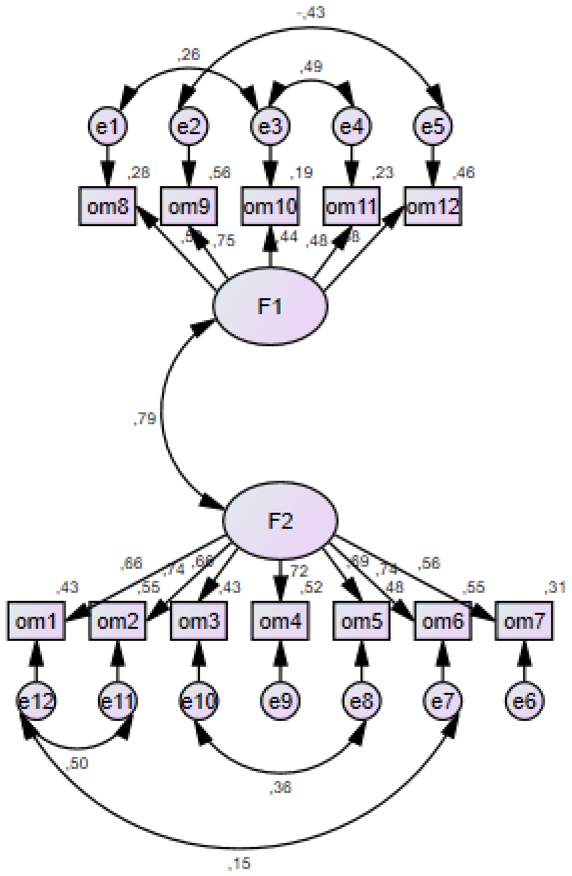

Supplement: Supplementary file 1 [file Data_Sheet_1.pdf]
